# Association between Sarcopenia and Depression in Patients with Chronic Liver Diseases

**DOI:** 10.3390/jcm8050634

**Published:** 2019-05-08

**Authors:** Hiroki Nishikawa, Hirayuki Enomoto, Kazunori Yoh, Yoshinori Iwata, Yoshiyuki Sakai, Kyohei Kishino, Naoto Ikeda, Tomoyuki Takashima, Nobuhiro Aizawa, Ryo Takata, Kunihiro Hasegawa, Noriko Ishii, Yukihisa Yuri, Takashi Nishimura, Hiroko Iijima, Shuhei Nishiguchi

**Affiliations:** Division of Hepatobiliary and Pancreatic disease, Department of Internal Medicine, Hyogo College of Medicine, Nishinomiya, Hyogo 663-8501, Japan; enomoto@hyo-med.ac.jp (H.E.); mm2wintwin@ybb.ne.jp (K.Y.); yo-iwata@hyo-med.ac.jp (Y.I.); sakai429@hyo-med.ac.jp (Y.S.); hcm.kyohei@gmail.com (K.K.); nikeneko@hyo-med.ac.jp (N.I.); tomo0204@yahoo.co.jp (T.T.); nobu23hiro@yahoo.co.jp (N.A.); chano_chano_rt@yahoo.co.jp (R.T.); hiro.red1230@gmail.com (K.H.); ishinori1985@yahoo.co.jp (N.I.); gyma27ijo04td@gmail.com (Y.Y.); tk-nishimura@hyo-med.ac.jp (T.N.); hiroko-i@hyo-med.ac.jp (H.I.); nishiguc@hyo-med.ac.jp (S.N.)

**Keywords:** chronic liver disease, sarcopenia, depression, beck depression inventory

## Abstract

Association between sarcopenia, as evaluated by grip strength (GS) and skeletal muscle mass (SMM), and depression, as evaluated by Beck Depression Inventory-2nd edition (BDI-II) in chronic liver diseases (CLDs, *n* = 414, average age = 61.5 years), was investigated. Study subjects were classified into four groups: Group A (*n* = 60), lower GS and lower SMM (sarcopenia); group B (*n* = 44), lower GS and higher SMM; group C (*n* = 100), higher GS and lower SMM; group D (*n* = 210), higher GS and higher SMM. Factors associated with BDI-II score ≥11 were examined. BDI-II score 0–10 (normal) was found in 284 (68.6%), 11–16 (minimal) in 76 (18.4%), 17–20 (mild) in 24 (5.8%), 21–30 (moderate) in 15 (3.6%), and ≥31 (severe) in 15 (3.6%). The average ± standard deviation BDI-II score in liver cirrhosis (LC) patients (10.2 ± 9.6, *n* = 152) was significantly higher than that in non-LC patients (7.4 ± 7.2, *n* = 262) (*p* = 0.0058). Univariate analysis identified three factors to be significantly associated with BDI-I score ≥11: Our classification (groups of A, B, C, and D) (*p* = 0.0259), serum albumin (*p* = 0.0445), and the presence of LC (*p* = 0.0157). Multivariate analysis revealed that only group A (*p* = 0.0074, group D as a reference) was significant. In conclusion, sarcopenia can be an independent predictor for depression in CLDs.

## 1. Introduction

Depression is common in patients with chronic liver diseases (CLDs) [1,2,3,4,5,6,7,8]. Even in CLD patients with an earlier liver fibrotic stage, depressive symptoms can be frequently observed compared with healthy persons [7]. In chronic hepatitis C patients, depression, anxiety, and fatigue are shown to be at the top of the list of psychiatric disorders [9]. Impaired quality of life (QOL) and increasing health care costs have been reported for CLD patients with depression [4,10]. In liver cirrhosis (LC) patients, depression can be associated with substantial morbidity and mortality, however, it is under-diagnosed and this topic is not given full attention in current practice guidelines [1]. The Beck Depression Inventory-2nd edition (BDI-II) is one of the most extensively used screening tools for depression and its usefulness has been well validated [11].

Sarcopenia is a clinical sign characterized by diminished skeletal muscle mass (SMM) and poor muscle function, resulting in frailty, cachexia, osteoporosis, and thereby all-cause mortality [12,13,14,15,16,17,18,19,20,21,22,23,24,25]. The pathophysiology of sarcopenia includes daily life inactivity, bedridden condition, poor nutrition, hormone deficiency and chronic inflammation [26]. Alterations in nutritional metabolism, nutritional requirements, and reduced dietary intake are common in CLDs and sarcopenia can also be linked to worse patient QOL, poor prognosis, and higher health care costs in CLDs [15,17,19]. Sarcopenia can be a contributor of malnutrition or a consequence of malnutrition and primarily responsible for the unfavorable clinical consequences observed in CLD patients [15,17,19]. In Japan, 10–70% of CLD patients have sarcopenia. This prompted the Japan Society of Hepatology (JSH) to create guidelines for the diagnosis of sarcopenia in liver disease (first edition) in 2016 [14,18]. The JSH criteria adopts grip strength (GS) for the evaluation of muscle strength and bioimpedance analysis (BIA) and/or computed tomography for the evaluation of SMM, while unlike the Asian criteria for sarcopenia, age restriction (i.e., 65 years old or more) is not set in the JSH criteria for the assessment of sarcopenia because CLD patients can present diminished SMM due to impaired protein synthesis regardless of age [14,18].

However, data for the relevance between sarcopenia and depression in CLD patients remain limited, although recent studies demonstrated that sarcopenia was independently associated with depression in middle-aged or elderly individuals [27,28,29,30,31]. In the current study, we sought to investigate whether an independent association exists between sarcopenia and depression, as evaluated by BDI-II in patients with CLDs.

## 2. Experimental Section

### 2.1. Patients

A total of 435 CLD patients with data for GS, SMM using BIA and the BDI-II available were admitted to our hospital between November 2013 and August 2018. Fifteen patients with underlying diseases such as overt hepatic encephalopathy, advanced malignancies, severe inflammatory diseases, or liver-unrelated psychiatric diseases were excluded because they potentially affected the interpretation of BDI-II score. As overestimates could occur for the calculation of skeletal muscle mass index (SMI) using BIA in patients with massive ascites, 6 subjects with severe ascites were also excluded from the study [15]. A total of four-hundred and fourteen patients were thus analyzed. The diagnosis of LC was based on laboratory data, radiographic findings (presence or absence of varices, deformity of the liver or ascites), and/or liver biopsy data (F4 or not).

### 2.2. Questionnaire Survey

The BDI-II is a globally used screening tool for evaluating the severity of depression [32,33]. The BDI-II has good psychometric properties, high reliability and internal consistency and it is a self-administered questionnaire that comprises 21 items, and individual answers are evaluated on a four-point scale (0 to 3 points) [32,34]. Higher BDI-II scores suggest a more severe depression state. Our study subjects were categorized as normal (BDI-II score, 0–10), and the severity of depression state as minimal (BDI-II score, 11–16), mild (BDI-II score, 17–20), moderate (BDI-II score, 21–30), and severe (BDI-II score ≥31) [33,35,36,37].

### 2.3. Measurement of GS and SMI and Our Study

GS was measured according to the current guidelines [14]. Two measurements of GS were done on both the left and right sides. The better measurement on each side is utilized, and GS was calculated as the mean values of these. SMI was defined as “appendicular SMM/height squared (m^2^)” by BIA. Based on the current guidelines, patients with lower GS (L-GS) were defined as those with GS less than 26 kg for men and less than 18 kg for women and those with higher GS (H-GS) were defined as those with GS 26 kg or more for men and 18 kg or more for women [14]. Likewise, patients with lower SMM (L-SMM) were defined as those with SMI)less than 7.0 kg/m^2^ for men and less than 5.7 kg/m^2^ for women and those with higher SMM (H-SMM) were defined as those with SMI 7.0 kg/m^2^ or more for men and 5.7 kg/m^2^ or more for women [14]. (Table 1) In male, patients with GS less than 26 kg and SMI less than 7.0 kg/m^2^ were classified as group A (sarcopenia), those with GS less than 26 kg and SMI 7.0 kg/m^2^ or more as group B, those with GS 26 kg or more and SMI less than 7.0 kg/m^2^ as group C, and those with GS 26 kg or more and SMI 7.0 kg/m^2^ or more as group D. In female, patients with GS less than 18 kg and SMI less than 5.7 kg/m^2^ were classified as group A, those with GS less than 18 kg and SMI 5.7 kg/m^2^ or more as group B, those with GS 18 kg or more and SMI less than 5.7 kg/m^2^ as group C, and those with GS 18 kg or more and SMI 5.7 kg/m^2^ or more as group D. 

Impacts of GS and SMM on depression state as evaluated by BDI-II were investigated for all cases and several subgroups according to the LC status, gender and age. BDI-II scores among 4 groups (group A, B, C and D) were also compared. Next, factors associated with BDI-II score ≥11 (minimal, mild, moderate or severe depression state) were examined using univariate and multivariate analyses. We received the ethical approval from ethics committee of our hospital (approval no, 2296). The protocol in the study strictly observed all regulations of the Declaration of Helsinki. 

### 2.4. Statistical Considerations

As for continuous parameters, Student’s *t* test, Mann-Whitney *U* test, Pearson’s correlation test, analysis of variance or Kruskal-Wallis test were employed to estimate between-group difference, as applicable. In categorical parameters, Fisher’s exact tests or Pearson χ^2^ test was employed to estimate between-group difference, as applicable. Factors with *p* < 0.05 linked to BDI-II score ≥11 in the univariate analysis were subjected to the multivariate logistic regression analysis to identify candidate parameters. Unless otherwise stated, data are presented as average ± standard deviation (SD) (interquartile range (IQR)). The threshold for statistical significance was set at *p* < 0.05. The JMP 13.2 (SAS Institute Inc., Cary, NC, USA) was employed to carry out statistical analysis.

## 3. Results

### 3.1. Patient Baseline Characteristics

Baseline characteristics in our study (n = 414, 198 males and 216 females, average ± SD (IQR) age = 61.5 ± 12.7 (52, 71) years) are presented in Table 2. The average ± SD (IQR) BDI-II score was 8.4 ± 8.3 [2,13]. BDI-II score 0–10 (normal) was found in 284 (68.6%), 11–16 (minimal depression) in 76 (18.4%), 17–20 (mild depression) in 24 (5.8%), 21–30 (moderate depression) in 15 (3.6%) and ≥31 (severe depression) in 15 (3.6%) [32]. Depression with BDI-II score ≥11 was thus observed in 130 patients (31.4%). Sarcopenia (i.e., group A), as defined by the JSH criteria, was found in 26 male patients (13.1%) and 34 female patients (15.7%) [14]. Among groups of A (*n* = 60), B (*n* = 44), C (*n* = 100), and D (*n* = 210), overall differences were identified with statistical significance in terms of age (*p* < 0.0001), gender (*p* = 0.0019), presence of LC (*p* = 0.0007), body mass index (BMI) (*p* < 0.0001), serum albumin (*p* < 0.0001), prothrombin time (*p* = 0.0004), platelet count (*p* = 0.0013), total cholesterol (*p* = 0.0085), estimated glomerular filtration rate (*p* = 0.0495), and BDI-II score (*p* < 0.0001). There were 152 LC patients (36.7%, Child-Pugh A in 113, Child-Pugh B in 37 and Child-Pugh C in 2). The average ± SD (IQR) BDI-II score in LC patients (10.2 ± 9.6 (3, 13.75)) was significantly higher than that in non-LC patients (7.4 ± 7.2 (2, 12)) (*p* = 0.0058), suggesting the more serious depression state in LC patients vs. non-LC patients. FIB-4 index significantly correlated with BDI-II score (*r* = 0.100, *p* = 0.0413). In our study subjects, there were no sarcopenic obesity patients as defined by sarcopenia and BMI >30 kg/m^2^ [12,15,17]. At baseline, 82 (31.9%) out of 257 patients with hepatitis C virus (HCV) had undetectable HCV-RNA. The BDI-II score in patients with undetectable HCV-RNA was significantly lower than that in patients without undetectable HCV-RNA (average ± SD: 6.9 ± 7.4 vs. 9.7 ± 8.7, *p* = 0.0116). At baseline, 37 (60.7%) out of 61 patients with hepatitis B virus (HBV) had undetectable HBV-DNA. The BDI-II score in patients with undetectable HBV-DNA was not significantly lower than that in patients without undetectable HBV-DNA (average ± SD: 8.1 ± 9.0 vs. 7.8 ± 7.4, *p* = 0.9055).

### 3.2. Impact of GS and SMM on BDI-II Score for All Cases (n = 414) 

**Figure 1:**
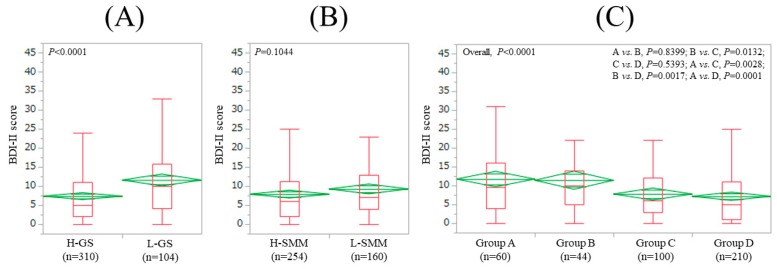
BDI-II score stratified by GS and SMM for all cases (*n* = 414). (**A**) Lower GS (L-GS) vs. Higher GS (H-GS). (**B**) Lower SMM (L-SMM) vs. Higher SMM (H-SMM). (**C**) Comparison among four groups (group A, B, C, and D). Group A indicates L-GS and L-SMM. Group B indicates L-GS and H-SMM. Group C indicates H-GS and L-SMM. Group D indicates H-GS and H-SMM.

The average ± SD (IQR) BDI-II score in the L-GS (11.6 ± 9.7(4.25, 15.75), *n* = 104) was significantly higher than that in the H-GS (7.4 ± 7.4 (2,11), *n* = 310) (*p* < 0.0001) (Figure 1A). The average ± SD (IQR) BDI-II score in the L-SMM (9.3 ± 8.3 (4, 13), *n* = 160) was similar to that in the H-SMM (7.9 ± 8.2 (2, 11.25), *n* = 254) (*p* = 0.1044) (Figure 1B). In comparison among four groups, the overall difference was observed with significance (*p* < 0.0001) (Figure 1C).

### 3.3. Impact of GS and SMM on BDI-II Score for LC Patients (n = 152) and Non-LC Patients (n = 262)

**Figure 2:**
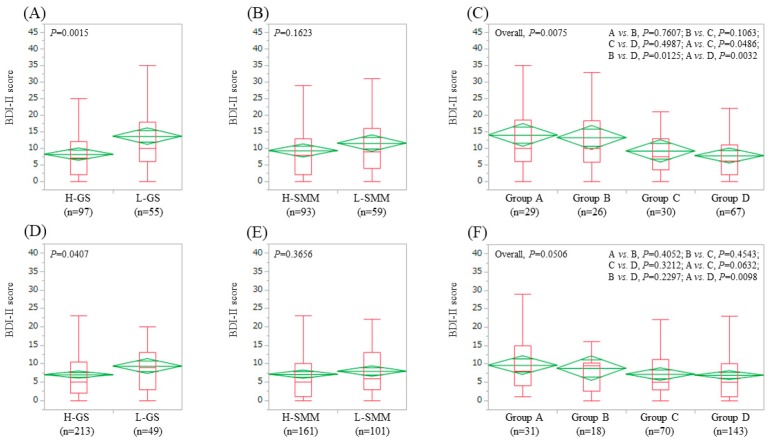
BDI-II score stratified by GS and SMM for LC cases (*n* = 152). (**A**) Lower GS (L-GS) vs. Higher GS (H-GS). (**B**) Lower SMM (L-SMM) vs. Higher SMM (H-SMM). (**C**) Comparison among four types (group A, B, C and D). BDI-II score stratified by GS and SMM for non-LC cases (*n* = 262). (**D**) L-GS vs. H-GS. (**E**) L-SMM vs. H-SMM. (**F**) Comparison among four groups (group A, B, C and D). Group A indicates L-GS and L-SMM. Group B indicates L-GS and H-SMM. Group C indicates H-GS and L-SMM. Group D indicates H-GS and H-SMM.

In LC patients, the average ± SD (IQR) BDI-II score in the L-GS (13.6 ± 11.1 (6, 18), *n* = 55) was significantly higher than that in the H-GS (8.2 ± 8.1 (2, 12), *n* = 97) (*p* = 0.0015) (Figure 2A). The average ± SD (IQR) BDI-II score in the L-SMM (11.5 ± 10.1 (4, 16), *n* = 59) was similar to that in the H-SMM (9.3 ± 9.2 (2, 13), *n* = 93) (*p* = 0.1623) (Figure 2B) In comparison among four groups, the overall difference was noted with significance (*p* = 0.0075) (Figure 2C).

In non-LC patients, the average ± SD (IQR) BDI-II score in the L-GS (9.3 ± 7.3 (3, 13), *n* = 49) was significantly higher than that in the H-GS (7.0 ± 7.1 (2, 10.5), *n* = 213) (*p* = 0.0407) (Figure 2D). The average ± SD (IQR) BDI-II score in the L-SMM (7.9 ± 6.6 (3, 13), *n* = 101) was identical to that in the H-SMM (7.1 ± 7.5 (1, 10), *n* = 161) (*p* = 0.3656) (Figure 2E). In comparison among four groups, the overall difference was noted with trend for significance (*p* = 0.0506) (Figure 2F).

### 3.4. Impact of GS and SMM on BDI-II Score for Male Patients (n = 198) and Female Patients (n = 216)

**Figure 3:**
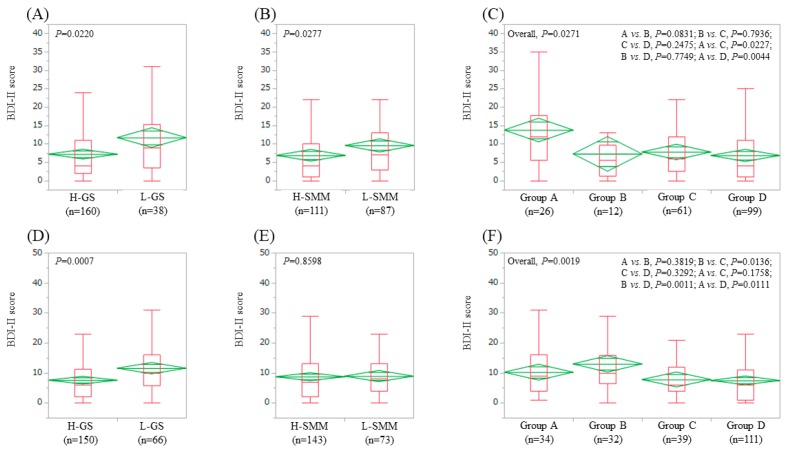
BDI-II score stratified by GS and SMM for male patients (*n* = 198). (**A**) Lower GS (L-GS) vs. Higher GS (H-GS). (**B**) Lower SMM (L-SMM) vs. Higher SMM (H-SMM). (**C**) Comparison among four types (group A, B, C and D). BDI-II score stratified by GS and SMM for female patients (*n* = 216). (**D**) L-GS vs. H-GS. (**E**) L-SMM vs. H-SMM. (**F**) Comparison among four groups (group A, B, C and D). Group A indicates L-GS and L-SMM. Group B indicates L-GS and H-SMM. Group C indicates H-GS and L-SMM. Group D indicates H-GS and H-SMM.

In male patients, the average ± SD (IQR) BDI-II score in the L-GS (11.7 ± 11.2 (3.5, 15.25), *n* = 38) was significantly higher than that in the H-GS (7.2 ± 7.5 (2, 11), *n* = 160) (*p* = 0.0220) (Figure 3A). The average ± SD (IQR) BDI-II score in the L-SMM (9.6 ± 9.5 (3, 13), *n* = 87) was significantly higher than that in the H-SMM (6.9 ± 7.5 (1, 10), *n* = 111) (*p* = 0.0277) (Figure 3B). In comparison among four groups, the overall difference was noted with significance (*p* = 0.0271) (Figure 3C).

In female patients, the average ± SD (IQR) BDI-II score in the L-GS (11.5 ± 8.8 (5.75, 16), *n* = 66) was significantly higher than that in the H-GS (7.5 ± 7.4 (2, 11.25), *n* = 150) (*p* = 0.0007) (Figure 3D). The average ± SD (IQR) BDI-II score in the L-SMM (8.9 ± 6.5 (4, 13), *n* = 73) was identical to that in the H-SMM (8.7 ± 8.8 (2, 13), *n* = 143) (*p* = 0.8598) (Figure 3E). In comparison among four groups, the overall difference was noted with significance (*p* = 0.0019) (Figure 3F).

### 3.5. Impact of GS and SMM on BDI-II Score for Patients Aged ≥65 Years (n = 199) and Patients Aged <65 Years (n = 215)

**Figure 4:**
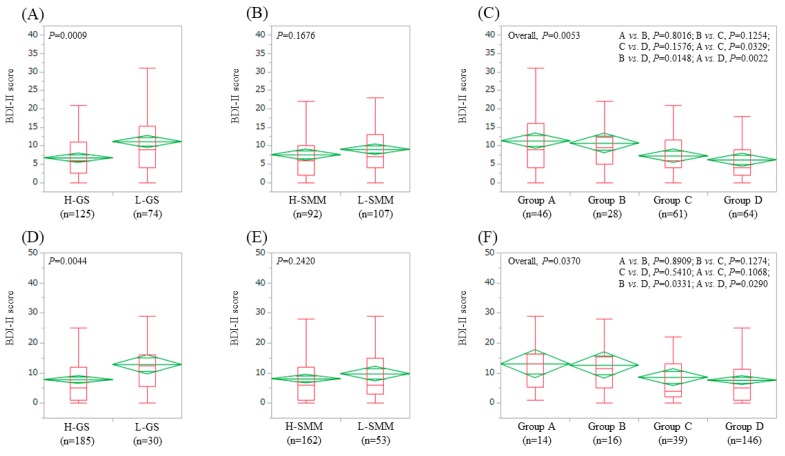
BDI-II score stratified by GS and SMM for patients >65 years (*n* = 199). (**A**) Lower GS (L-GS) vs. Higher GS (H-GS). (**B**) Lower SMM (L-SMM) vs. Higher SMM (H-SMM). (**C**) Comparison among four types (group A, B, C and D). BDI-II score stratified by GS and SMM for patients <65 years (*n* = 215). (**D**) L-GS vs. H-GS. (**E**) L-SMM vs. H-SMM. (**F**) Comparison among four groups (group A, B, C and D). Group A indicates L-GS and L-SMM. Group B indicates L-GS and H-SMM. Group C indicates H-GS and L-SMM. Group D indicates H-GS and H-SMM.

In patients aged ≥65 years, the average ± SD (IQR) BDI-II score in the L-GS (11.1 ± 9.4 (4, 15.25), *n* = 74) was significantly higher than that in the H-GS (6.7 ± 5.3 (2.5, 11), *n* = 125) (*p* = 0.0009) (Figure 4A). The average ± SD (IQR) BDI-II score in the L-SMM (9.0 ± 7.7 (4, 13), *n* = 107) was similar to that in the H-SMM (7.5 ± 7.1 (2, 10), *n* = 92) (*p* = 0.1676) (Figure 4B). In comparison among four groups, the overall difference was noted with significance (*p* = 0.0053) (Figure 4C)

In patients aged <65 years, the average ± SD (IQR) BDI-II score in the L-GS (12.8 ± 10.3 (5.5, 16), *n* = 30) was significantly higher than that in the H-GS (7.8 ± 8.6 (1, 12), *n* = 185) (*p* = 0.0044) (Figure 4D). The average ± SD (IQR) BDI-II score in the L-SMM (9.8 ± 9.4 (3, 15), *n* = 53) was identical to that in the H-SMM (8.1 ± 8.9 (1, 12), *n* = 162) (*p* = 0.2420) (Figure 4E). In comparison among four groups, the overall difference was noted with significance (*p* = 0.0370) (Figure 4F).

### 3.6. Univariate and Multivariate Analyses of Factors Associated with BDI-II Score ≥11

Univariate analysis identified three factors to be significantly associated with BDI-II score ≥11: our classification (groups of A, B, C and D) (*p* = 0.0259), serum albumin level (*p* = 0.0445) and the presence of LC (*p* = 0.0157). (Table 3) Multivariate analysis for the three factors (*p* < 0.05 in the univariate analyses) revealed that only group A (*p* = 0.0074, group D as a reference) was a significant factor linked to BDI-II score ≥11. (Table 4) Hazard ratios and 95% confidence intervals are listed in Table 3. 

### 3.7. Correlation between BDI-II Score and GS and SMI in Male and Female

In male, GS (*r* = −0.0973, *p* = 0.1976) and SMI (*r* = −0.0930, *p* = 0.1927) did not significantly correlate with BDI-II score. In female, GS significantly correlated with BDI-II score (*r* = −0.194, *p* = 0.006), while SMI did not (*r* = 0.0136, *p* = 0.8420). 

## 4. Discussion

The majority of CLD patients with depression remain under-diagnosed and there is growing interest for sarcopenia in CLDs. To examine the association between sarcopenia and depression in CLD patients is therefore clinically of great importance. Regarding reasons why the high prevalence of CLD patients with depression is observed, the following aspects should be considered: (a) The long-term suffering caused by CLD itself, worry about disease progression and fear of infection; (b) social pressure for studying and working; and (c) economic pressure including high cost for expected medical treatments such as antiviral therapies [5].

Our study findings revealed a significant association between sarcopenia and depression in CLD patients, independent of age, gender and LC status. These results were identical to the findings of previous longitudinal or cross-sectional studies on the association between depressive symptoms and sarcopenia. A recent prospective study (*n* = 691) demonstrated that the incidence of depressive symptoms increased with some chronic diseases including sarcopenia, type 2 diabetes mellitus and cardiovascular disease and SMM was the most relevant protective factor among SMM, muscle strength and physical performance [38]. A meta-analysis comprising 15 observational studies denoted that sarcopenic patients were likely to present with depressive symptoms [31]. Liver disease-related sarcopenia is a secondary sarcopenia due to chronic inflammation etc, while most previous studies on sarcopenia cover elderly persons because primary sarcopenia is a disease entity in an aging population [14,15,16,17,18]. In this respect, our study appears to be clinically of importance. On the other hand, patients with a depressive mood are likely to have daily life inactivity, which is a well-recognized cause of sarcopenia, and thus the close association between sarcopenia and depression is not so surprising [39].

Numerous studies also examined the relationship between SMM, GS, and depressive symptoms; however, the definitive conclusion has not been reached. A previous study reported that depression in elderly Koreans was closely linked to lower SMM and sarcopenia, especially in men [40]. Decline in GS was shown to be associated with a higher risk for incidence of depression [41]. Wu, et al. demonstrated that SMM and GS were both inversely associated with depressive symptoms in elderly Chinese [42]. While in our multivariate analyses, group B (L-GS and H-SMM) and group C (H-GS and L-SMM) were not significant linked to BDI-II score ≥11, although L-GS certainly affected BDI-II score reviewing Figure 1A, Figure 2A,D, Figure 3A,D and Figure 4A,D. It seems likely that at least several similar underlying mechanisms exist between sarcopenia and depressive symptoms. Regarding comparison of the effect of GS and SMM on depression, GS rather than SMM affected depression in this study. In our previous investigation, we reported that GS rather than SMM was associated with health-related QOL in CLD patients [43]. Poorer QOL can lead to daily life inactivity and subsequent depression. These observations may be linked to our current results.

Previous studies have found an increased prevalence of depressive symptoms in patients with metabolic disorders [44,45,46]. Depression is twice as frequent in diabetic patients compared with non-diabetic patients [46]. Psychiatric conditions are characterized by an increased risk of metabolic syndrome including dyslipidemia, obesity, hypertension, and hyperglycemia [45]. However, in our data, metabolic parameters such as HbA1c, BMI, and total cholesterol level were not significant factors associated with BDI-II ≥11. When the cutoff levels of BDI-II scores were set to a higher score (21 points or 31 points), similar results were obtained. In CLD patients, liver disease itself rather than metabolic disorders may affect depressive state.

LC places a substantial psychological burden. In our analysis, presence of LC was a significant factor related to BDI-II ≥11 in the univariate analysis, although did not reach significance in the multivariate analysis (*p* = 0.1101). As mentioned earlier, impact of psychological distress on LC patients is not given full attention in current practice guidelines [1]. Not only LC-related complications such as varices, ascites and hepatocellular carcinoma, but also psychological disorders should be emphasized in LC patients. Clinicians are encouraged to pay more attention to the psychological status of LC patients in their filed practice.

A previous Japanese study reported that in elderly functionally-independent and community-dwelling 1731 persons, sarcopenic obesity was closely linked to depression whereas either sarcopenia or obesity alone was not linked depression [47]. Sarcopenic obesity can be an adverse predictor for CLD patients [48]. While in our study, there were no sarcopenic obesity patients. To clarify whether sarcopenic obesity is associated with depression in CLD patients, further study is needed.

The BDI-II score in patients with undetectable HCV-RNA at baseline was significantly lower than that in patients without undetectable HCV-RNA in our current analysis. A sense of relief for the HCV being eradicated may be attributed to our results. On the other hand, eGFR was not significant in our univariate analysis. A recent study demonstrated that sarcopenia was associated with age and physical capacity, but not with eGFR or dietary intakes in chronic kidney disease patients [49]. While sarcopenia can be linked to depression in our data. Their reports may be associated with our results. 

We acknowledge several limitations to this study. First, our study was a single-center cross-sectional study with a retrospective nature. Second, BDI-II is a patient-reported subjective assessment method for depression and objective assessment was not done in this study. Third, GS can vary depending on patient daily life activities. Fourth, patients with massive ascites or overt hepatic encephalopathy who are potentially involved in liver disease-related sarcopenia were excluded due to the lack of reliability in BIA or patient-reported questionnaire, leading to bias. Lindqvist, et al. reported that dual-energy x-ray absorptiometry and computed tomography are useful for the calculation of muscle mass in CLD patients with pretransplant status irrespective of the presence of ascites [50]. Further studies using other alternative methods for muscle mass should be therefore performed. Fifth, clinical information for concomitant drug use, alcohol intake or social situation that can be associated with depression in each patient was not sufficient in the current analysis. Finally, it was unclear as to whether sarcopenia caused depression or vice versa in our study. Caution should be therefore exercised for the interpretation of our data. Nevertheless, our study results demonstrated that sarcopenia in CLDs was closely associated with depression. In conclusion, sarcopenia can be an independent predictor for depression in patients with CLDs.

## Figures and Tables

**Table 1:** Cut-off values of bioimpedance analysis and grip strength according to gender.

Modality	Cut-off Value
Bioimpedance analysis	Male: 7.0 kg/m²
	Female: 5.7 kg/m²
Grip strength	Male: 26 kg
	Female: 18 kg

**Table 2:** Baseline characteristics.

Variables	All Cases (*n* = 414)	Group A (*n* = 60)	Group B (*n* = 44)	Group C (*n* = 100)	Group D (*n* = 210)	Overall *p* Value
**Age (years)**	61.5 ± 12.7 (52, 71)	68.3 ± 12.2 (65, 75)	65.9 ± 10.7 (63, 73)	64.6 ± 12.2 (60, 72)	57.1 ± 11.8 (48, 66)	<0.0001
**Gender, male/female**	198/216	26/34	12/32	61/39	99/111	0.0019
**HBV/HCV/HBV and HCV/NBNC (Etiology)**	61/257/8/88	5/41/2/12	7/27/0/10	15/67/3/15	34/122/3/51	0.4858
**Presence of LC, yes/no**	152/262	29/31	26/18	30/70	67/143	0.0007
**Body mass index (kg/m^2^)**	23.3 ± 3.8 (20.6, 25.5)	21.2 ± 2.2 (19.8, 22.7)	25.8 ± 3.8 (22.9, 28.8)	20.8 ± 2.5 (19.0, 22.1)	24.6 ± 3.8 (21.9, 26.7)	<0.0001
**Total bilirubin (mg/dL)**	0.98 ± 0.60 (0.6, 1.1)	0.9 ± 0.4 (0.6, 1.0)	1.3 ± 0.9 (0.6, 1.7)	0.9 ± 0.4 (0.7, 1.0)	1.0 ± 0.6 (0.6, 1.1)	0.1482
**Serum albumin (g/dL)**	4.1 ± 0.5 (3.9, 4.5)	3.9 ± 0.6 (3.5, 4.4)	3.9 ± 0.6 (3.5, 4.2)	4.3 ± 0.4 (4.1, 4.6)	4.2 ± 0.5 (4.0, 4.5)	<0.0001
**Prothrombin time (%)**	86.4 ± 15.9 (79.3, 96.5)	85.4 ± 16.6 (77.7, 94.4)	77.3 ± 16.5 (62.6, 89.5)	86.9 ± 16.2 (82.0, 96.3)	88.4 ± 14.9 (80.2, 98.6)	0.0004
**Platelet count (×10^4^/mm^3^)**	17.0 ± 7.1 (11.7, 21.2)	16.6 ± 7.4 (10.4, 21.2)	13.3 ± 6.7 (7.4, 17.9)	16.7 ± 6.0 (12.1, 20.1)	18.0 ± 7.4 (12.6, 22.5)	0.0013
**Total cholesterol (mg/dL)**	181.1 ± 41.7 (150, 207.5)	177.4 ± 53.2 (143, 209)	161.8 ± 38.2 (135, 186)	184.1 ± 38.5 (157, 206)	184.7 ± 39.3 (154, 212)	0.0085
**AST (IU/L)**	36.9 ± 26.5 (21, 42)	41.4 ± 31.1 (21, 49)	41.4 ± 27.0 (22, 48)	35.0 ± 23.4 (22, 37)	35.5 ± 26.3 (21, 40)	0.2554
**ALT (IU/L)**	36.1 ± 35.7 (16, 44)	37.3 ± 43.9 (15, 42)	37.7 ± 37.1 (16, 43)	33.3 ± 28.4 (16, 40)	36.7 ± 36.1 (16, 44)	0.8485
**ALP (IU/L)**	284.1 ± 274.2 (195, 300)	377.4 ± 640.4 (206, 343)	281.2 ± 110.9 (215, 314)	253.1 ± 93.6 (195, 279)	272.2 ± 141.8 (192, 298)	0.2601
**GGT (IU/L)**	47.3 ± 58.7 (17, 49)	48.4 ± 69.6 (16, 47)	54.6 ± 56.2 (21, 58)	44.4 ± 54.5 (17, 46)	46.8 ± 58.1 (17, 49)	0.8193
**eGFR mL/min/1.73 m^2^)**	83.6 ± 21.4 (72.0, 95.5)	78.6 ± 24.1 (65.8, 90.5)	82.4 ± 25.5 (71, 93)	81.4 ± 19.7 (71, 90)	86.3 ± 20.1 (74, 99)	0.0495
**HbA1c (NGSP)**	5.8 ± 0.8 (5.4, 6.0)	5.8 ± 0.8 (5.3, 6.0)	5.8 ± 0.9 (5.2, 6.4)	5.8 ± 0.8 (5.5, 6.0)	5.8 ± 0.8 (5.4, 6.0)	0.9614
**Serum sodium (mmol/L)**	140.1 ± 2.4 (139, 142)	139.6 ± 3.2 (138, 142)	139.9 ± 2.8 (139, 142)	140.4 ± 2.3 (140, 142)	140.1 ± 2.2 (139, 141)	0.1840
**BDI-II score**	8.4 ± 8.3 (2, 13)	11.7 ± 9.6 (4, 16)	11.4 ± 9.9 (5, 14)	7.8 ± 7.0 (3, 12)	7.2 ± 7.7 (1, 11)	<0.0001

Data are expressed as average ± standard deviation (interquartile range). HBV; hepatitis B virus, HCV; hepatitis C virus, NBNC; non-B and non-C, LC; liver cirrhosis, AST; aspartate aminotransferase, ALT; alanine aminotransferase, ALP; alkaline phosphatase, GGT; gamma-glutamyltransferase, eGFR; estimated glomerular filtration rate, NSGP; National Glycohemoglobin Standardization Program, BDI-II; Beck depression inventory II.

**Table 3:** Univariate analyses of factors linked to BDI-II score ≥11.

Variables	BDI-II Score ≥11 (*n* = 130)	BDI-II Score <11 (*n* = 284)	*p* Value
**Age (years)**	60.3 ± 13.5 (50.75, 70.25)	62.0 ± 12.3 (54, 71)	0.2064
**Gender, male/female**	61/69	137/157	0.8326
**HBV/HCV/HBV and HCV/NBNC**	17/86/2/25	44/171/6/63	0.7106
**Body mass index (kg/m^2^)**	23.2 ± 3.9 (20.3, 25.025)	23.3 ± 3.8 (20.625, 25.6)	0.6580
**Presence of LC, yes/no**	59/71	93/191	0.0157
**Group, A/B/C/D**	28/16/30/56	32/28/70/154	0.0259
**Total bilirubin (mg/dL)**	0.95 ± 0.55 (0.6, 1.1)	0.99 ± 0.62 (0.6, 1.0)	0.5333
**Serum albumin (g/dL)**	4.05 ± 0.54 (3.8, 4.4)	4.15 ± 0.52 (3.9, 4.5)	0.0445
**Prothrombin time (%)**	85.9 ± 15.8 (77.65, 96.375)	86.6 ± 16.0 (79.625, 97.1)	0.6875
**Platelet count (×10^4^/mm^3^)**	16.4 ± 7.0 (10.975, 20.625)	17.2 ± 7.2 (11.95, 21.8)	0.2924
**AST (IU/L)**	39.5 ± 26.6 (21, 48)	35.7 ± 26.4 (21, 39.75)	0.1703
**ALT (IU/L)**	39.2 ± 40.5 (16, 47.25)	34.6 ± 33.2 (16, 42)	0.2200
**ALP (IU/L)**	278.7 ± 140.0 (200, 305.5)	286.5 ± 317.6 (193.25, 299.75)	0.7936
**GGT (IU/L)**	52.1 ± 69.4 (21, 50)	45.0 ± 52.9 (17, 49)	0.2630
**Total cholesterol (mg/dL)**	176.6 ± 39.6 (145, 204.25)	183.1 ± 42.6 (151.75, 211)	0.1492
**eGFR (mL/min/1.73 m^2^)**	84.8 ± 20.8 (73, 96)	83.0 ± 21.6 (71, 95)	0.4335
**Serum sodium (mmol/L)**	140.0 ± 2.9 (139, 142)	140.1 ± 2.2 (139, 142)	0.6158
**HbA1c (NGSP)**	5.8 ± 0.8 (5.3, 6.0)	5.8 ± 0.8 (5.4, 6.075)	0.8769

Data are expressed as average ± standard deviation (interquartile range). BDI-II; Beck depression inventory II, HBV; hepatitis B virus, HCV; hepatitis C virus, NBNC; non-B and non-C, LC; liver cirrhosis, AST; aspartate aminotransferase, ALT; alanine aminotransferase, ALP; alkaline phosphatase, GGT; gamma-glutamyltransferase, eGFR; estimated glomerular filtration rate, NSGP; National Glycohemoglobin Standardization Program.

**Table 4:** Multivariate analyses of factors linked to BDI-II score ≥11.

	Multivariate Analysis		
	Hazard Ratio	95% CI	*p* Value
**Presence of LC**			
Yes	0.658	0.394–1.099	0.1101
No	1.000	Reference	
**Serum albumin (per 1.0 g/dL)**	1.027	0.641–1.646	0.910
**Group**			
A (L-GS and L-SMM)	0.438	0.239–0.801	0.0074
B (L-GS and H-SMM)	0.705	0.349–1.423	0.3289
C (H-GS and L-SMM)	0.813	0.477–1.385	0.4460
D (H-GS and H-SMM)	1.000	Reference	

BDI-II; Beck depression inventory II, LC; liver cirrhosis, L-GS; Lower grip strength, L-SMM; Lower skeletal muscle mass, H-GS; Higher grip strength, H-SMM; Higher skeletal muscle mass, CI; confidence interval.

## Abbreviation

CLD; chronic liver disease, QOL; quality of life, LC; liver cirrhosis, BDI-II; Beck Depression Inventory-2nd edition, SMM; skeletal muscle mass, JSH; Japanese Society of Hepatology, GS; grip strength, BIA; bioimpedance analysis, SMI; skeletal muscle mass index, L-GS; lower grip strength, H-GS; higher grip strength, L-SMM; lower skeletal muscle mass, H-SMM; higher skeletal muscle mass, SD; standard deviation, IQR; interquartile range, HCV; hepatitis C virus, HBV; hepatitis B virus.
